# Biochemical, Antioxidant, and Antimicrobial Profiling of Essential Oils of Indian Origin for Culinary Applications

**DOI:** 10.1155/ijfo/9326683

**Published:** 2024-12-27

**Authors:** K. Sathish Kumar, S. Visnuvinayagam, G. Teena, K. Elavarasan, J. Bindu, Amjad K. Balange, R. Sivaranjani, L. Narasimhamurthy

**Affiliations:** ^1^Fish Processing Division, ICAR-Central Institute of Fisheries Technology, Cochin, Kerala, India; ^2^Fisheries Resources, Harvest & Post-Harvest Management Division, ICAR-Central Institute of Fisheries Education, Mumbai, Maharashtra, India; ^3^Microbiology, Fermentation and Biotechnology Division, ICAR-Central Institute of Fisheries Technology, Cochin, Kerala, India; ^4^Crop Production and Post-Harvest Technology, ICAR-Indian Institute of Spices Research, Kozhikode, Kerala, India; ^5^National Fisheries Development Board, Hyderabad, Telangana, India

**Keywords:** antimicrobial, antioxidant, chemical profile, essential oils (EOs), natural preservative

## Abstract

This study investigated the biochemical composition and tested the antioxidant and antimicrobial properties of four Indian-origin essential oils (EOs)—ginger, garlic, clove, and eucalyptus—to evaluate their potential for culinary applications. Gas chromatography–mass spectrometry (GC–MS) analysis was used to identify the chemical constituents of EOs. Antioxidant assays such as 2,2-diphenyl-1-picrylhydrazyl (DPPH) and ferric reducing antioxidant power (FRAP) and antimicrobial assays such as Agar well diffusion, minimum inhibitory concentration (MIC), and minimum bactericidal concentration (MBC) were carried out. In GC–MS analysis, *α*-zingiberene (28.01%), eugenol (70.12%), 1,8-cineole (52.95%), and allyl polysulfides and terpenes were the most dominant compounds in ginger, clove, eucalyptus, and garlic EOs, respectively, which are responsible for their antioxidant and antimicrobial properties. Based on the antioxidant assays, clove EO exhibited the strongest antioxidant activities in both DPPH (70.84 ± 3.95%) and FRAP (142.29 ± 1.05 *μ* mol Fe (II) g^−1^) even at 5% level, suggesting its potential to inhibit lipid peroxidation, a process linked to oxidative stress in food. The antimicrobial study demonstrated the potential of EOs against foodborne pathogens, particularly against methicillin-resistant *S. aureus* (MRSA), which reveals their potential against multidrug-resistant bacteria. Among them, clove EO demonstrated the most potent antimicrobial activity against both Gram-negative and Gram-positive bacteria, with notable activity against MRSA with an inhibition zone of 41.33 ± 0.57 mm. This strong antimicrobial activity of clove EO was directly correlated with its total phenolic content (375.91 ± 14.21 mg phenols 100 g^−1^ at 5% level). The results indicated that clove EO stands out for its strong antioxidant and antimicrobial properties, particularly against multidrug-resistant pathogens like MRSA. These findings suggest clove EO could be a promising natural alternative to synthetic preservatives and antibiotics in culinary applications, helping to preserve food and combat resistant bacteria.

## 1. Introduction

Recently, foodborne-related diseases are the most common health concern for humans worldwide. Microbial deterioration and oxidative rancidity are the two major factors for spoilage of foods [1]. During the 20th century, several new technologies and chemical compounds were developed for food preservation. However, pathogenic microorganisms in the food system continue to create multiple human diseases and cause substantial economic losses to food processors [2]. At the same time, the indiscriminate use of antibiotics in human medicines, animal husbandry, and food industries against pathogenic bacteria has led to antimicrobial-resistant (AMR) development [3, 4]. AMR continues to develop more powerful mechanisms than ever [5] and will be the major challenge shortly [6]. Globally, AMR gets much attention among the research communities since it is widespread, reduces the therapeutic effect of these drugs within a shorter period, and leads to severe problems in the entire healthcare system [7]. Therefore, AMR has been categorized as one of the top 10 public health threats to humans [8]. Fatality rates due to AMR infections are increasing drastically, and it is expected to reach nearly 10 million per year by 2050. If no more effective and unique therapeutic methods are identified to contain AMR, there will be tremendous global economic losses [9]. This situation forced the research communities to look back into traditional medicines or herbal products, which may provide appropriate and acceptable alternative solutions to maintain human health [10].

Oxidative rancidity is another factor for food spoilage during raw material handling, processing, and storage, which results in loss of nutritional quality, flavour, taste, and colour and leads to consumer rejections [11, 12]. Oxidized molecules in food systems are the causative agents for degenerative diseases such as arthritis, cancer, diabetes, multiple sclerosis, and cardiovascular-related diseases [13, 14]. Moreover, oxidation and rancidity are the biggest problems for the food industries, as they are directly involved in food waste generation and economic losses [12]. To avoid these situations, synthetic additives have been widely used in industries due to their low cost, purity, and high activity at low concentrations. However, some harmful effects such as allergies, poisoning, and metabolic disorders have been reported when used in high doses [15, 16]. Therefore, it is necessary to find new antioxidant compounds that can be added to foods without affecting consumers' health. Recently, natural antioxidants have received greater attention in the research communities to prevent oxidative rancidity in food products. It could be one of the best options for food industries to replace synthetic antioxidants or preservatives, due to its bioavailability and health-beneficial effects. In addition, consumers' awareness of quality food products processed with natural preservatives has forced the food industries to focus on new natural preservatives in recent times. As a result, finding a new preservative with antioxidant and antimicrobial properties from natural sources is a primary goal for several researchers to benefit food processors and consumers.

India is the land of spices and herbs and has made a major contribution to world production. Wide ranges of spices are produced in India, with varying tropical climate conditions that make almost all spices grow splendidly. During 2021–2022, spices/spice products export reached around 1,531,154 tons, valued at $4102.29 million [17]. Most spice extracts are in the form of essential oils (EOs). They are extensively used in folk medicines, food flavourings, and pharmaceutical applications. In addition, food industries use them as a natural preservative to extend storage stability without affecting the organoleptic properties [18–20], due to their unique properties, including antimicrobial, anti-inflammatory, antioxidant, and antitumour [21–23]. Some EOs are branded as generally recognized as safe (GRAS) by the Food and Drug Administration (FDA) and are considered potential alternatives to conventional antibiotics [24, 25]. However, a wide range of spices are cultivated in India, and their biofunctional properties and composition may vary due to several factors, such as variety, geographical conditions, maturity stage, and extraction methods, compared to worldwide production. In addition, the information on the antioxidant and antimicrobial component(s) of different Indian-origin EOs and their chemical compositions is minimal. Therefore, the current study is aimed at profiling the phytochemical composition and dual preservative properties, namely, antioxidant and antimicrobial properties, of four important commercial Indian-origin EOs, including clove, ginger, garlic, and eucalyptus.

## 2. Materials and Methods

### 2.1. Chemicals and Reagents

EOs such as clove, ginger, garlic, and eucalyptus were purchased from the local market. All the chemicals and reagents used as analytical research (AR) grade were obtained from Sigma–Aldrich, Merck, Hi-Media, and Qualigens.

### 2.2. Characterization of EOs by Gas Chromatography–Mass Spectrometry (GC–MS) Analyses

The chemical profile of EOs was carried out using GC–MS analysis (Model No. QC2010, Shimadzu, Japan) at ICAR-Indian Spice Research Institute, Kozhikode, India. For analysis, Restex 5MS capillary column having a length of 30 m × 0.25 mm inner diameter (ID), 0.25 *μ* of film thickness was used. The injector temperature was set at 250°C. GC oven temperature was programmed as follows: Initial holding temperature was kept at 60°C for 5 min, followed by heating temperature at 200°C for 3°C/min, and finally kept the constant temperature at 220°C for 7 min. The running time was set for 50 min, and Helium gas was used as a carrier gas with a steady flow speed (1.0 mL/min), and EOs (0.20 *μ*L) were injected into the instrument. An electron ionisation system with 70 eV ionisation voltage and a mass scanning range of 40–650 m/z was used for MS detection. The EO components were identified by comparing them with the NIST library or mass spectra from the literature [26].

### 2.3. In Vitro Antioxidant Activities of EOs

#### 2.3.1. 2,2-Diphenyl-1-picrylhydrazyl (DPPH) Free Radical Scavenging Capacity

The DPPH radical scavenging activity of EOs was measured according to Yen and Wu [27], with certain modifications. Briefly, an aliquot of 100 *μ*L EOs with various concentrations (20%–100% of ginger, garlic, and eucalyptus EOs and 1%–5% in clove EO) was added into 2 mL of methanolic DPPH (6 × 10^−5^ M). Afterward, the mixtures were vortexed (Neuation Digital Vortex Mixer, iSwix VT, India) and kept in a dark place for 30 min at room temperature. Finally, absorbance was measured at 517 nm using a UV-VIS spectrophotometer (UV-2600, Shimadzu, Kyoto, Japan). Methanolic DPPH solution was used as a control. The percentage inhibition was calculated using the following formula:

 $$\begin{array}{c} \%\text{I}=\frac{\left[\left({\text{A}}_{\text{control}}–{\text{A}}_{\text{sample}}\right)\right]}{\left[{\text{A}}_{\text{control}}\right]}\times 100 \end{array}$$

where *I* is the inhibition percentage of DPPH, *A*_control_ is the absorbance of DPPH solution at 0 min, and *A*_sample_ is the absorbance of DPPH solution with EOs after 30 min.

#### 2.3.2. Ferric Reducing Antioxidant Power (FRAP) Activity

The ferric-reducing capacity of different EOs was determined according to Benzie and Strain [28]. Briefly, 20 *μ*L of EO with various concentrations (20%–100% of ginger, garlic, and eucalyptus EOs and 1%–5% in clove EO) were mixed into freshly prepared FRAP reagent (2 mL). Then, the mixture was mixed well and kept in a dark place for 4 min. Subsequently, the absorbance at 593 nm was measured using a UV-VIS spectrophotometer (UV-2600, Shimadzu, Kyoto, Japan). FRAP reagent without EO was used as control. The obtained absorbance values were interpolated in a standard curve developed using iron (II) sulfate solution (100–2000 *μ*M). The results were expressed as micromole Fe (II)/grams.

#### 2.3.3. Total Phenolic Content (TPC)

The TPC of EOs was calculated according to Singleton and Rosy [29]. Briefly, 30 *μ*L of EOs with various concentrations (5%–25% ginger, garlic, and eucalyptus EOs and 1%–5% clove EO) were added into 3 mL distilled water and vortexed. Subsequently, the Folin–Ciocalteu reagent (0.5 mL) was mixed into the diluted EOs. After 3 min of incubation, 20% sodium carbonate (2 mL) was poured into the mixture and placed in a water bath for 1 min. Finally, absorbance at 650 nm was measured using a UV-VIS spectrophotometer (UV-2600, Shimadzu, Kyoto, Japan). Catechol is used as a standard to plot the standard curve. The phenolic content was catechol equivalents in mg phenols/100 g of EOs.

### 2.4. In Vitro Antimicrobial Activities of EOs

#### 2.4.1. Microorganisms and Culture

The bacterial strains—*Staphylococcus aureus* (ATCC: 25923), methicillin-resistant *Staphylococcus aureus* (MRSA) (ATCC: 43300), *Bacillus cereus* (lab isolate), *Salmonella enterica typhi* (ATCC 9150), *Vibrio cholera* (ATCC: 14033), *Escherichia coli* (ATCC: 10536), *Pseudomonas aeruginosa* (ATCC: 10145), and *Aeromonas hydrophila* (ATCC: 35654)—were procured from Microbial Type Culture Collection and Gene Bank (MTCC), Chandigarh, India. All the cultures were conserved at 4°C and kept alive by continuous subculture on a nutrient agar medium.

For the antimicrobial study, brain heart infusion (BHI) broth was inoculated with test bacterial culture, which was then cultured for 24 h at 37°C. Normal saline was used to adjust the bacterial culture turbidity (1.5 × 10^8^CFU/mL) with 0.5 McFarland standard turbidity.

#### 2.4.2. Agar Well Diffusion Method

The antibacterial properties of EOs were employed with the agar well diffusion method [30] against food-borne and fish-borne bacteria such as *S. aureus*, MRSA, *B. cereus*, *S. typhi*, *V. cholera*, *E. coli*, *P. aeruginosa*, and *A. hydrophila*. Briefly, the bacterial cell suspension (1.5 × 10^8^CFU/mL) was spread over Mueller–Hinton agar (MHA) (HiMedia, India) plates and followed by 6 mm diameter wells prepared using sterile cork borer. Subsequently, 100 *μ*L of EOs and the control were poured into each empty well. Then, the plates were incubated at 37°C for 24 h. Finally, the inhibition zone (*n* = 4) was measured using a standard antibiotic zone scale (PW 096, Hi Media).

#### 2.4.3. Evaluation of Minimum Inhibitory Concentration (MIC) and Minimum Bactericidal Concentration (MBC)

Resazurin microdilution assay was carried out to measure the MIC values [31] of EOs against food-borne and fish-borne bacterial strains such as *S. aureus*, MRSA, *B. cereus*, *S. typhi*, *V. cholera*, *E. coli*, *P. aeruginosa*, and *A. hydrophila*. In summary, an aliquot of 100 *μ*L Mueller–Hinton broth (MHB) (HiMedia, India) was filled into 96 well plates and followed by 200 *μ*L of EOs and 20 *μ*L of Tween 20 as an emulsifying/solubilizing agent was added into the first well. Further, serial dilution (100% to lower level) was carried out until the last well. At last, 30 *μ*L (approx. 3.0 × 10^6^CFU/mL) of bacterial suspension (0.5 McFarland standard adjusted) was added to each well and mixed thoroughly. After overnight incubation at 37°C, resazurin solution (30 *μ*L) was added into each well and incubated further at 37°C for 1 h. The MIC values are defined as the lowest concentration before the colour change. To determine MBC, a loopful of inoculum was taken from wells before the colour change and streaked on MHA plates. Then, the plates were incubated for 24 h at 37°C. The lowest concentration, which has no bacterial growth on the MHA plates, was considered MBC.

### 2.5. Statistical Analyses

The results are expressed in mean ± standard deviation (*n* = 3). The variance among the samples was analyzed using a one-way analysis of variance (ANOVA), and a comparison of mean values was performed by Duncan's multiple range test (*p* ≤ 0.05).

## 3. Results and Discussion

### 3.1. Chemical Constituents of EOs

The chemical constituents of ginger, clove, eucalyptus, and garlic EOs with retention time and percentage share in the sample are represented in Tables 1 and 2. About 31 components were identified from ginger EO, comprising more than 94% of the EO composition (Table 1). The most abundant compounds included *α*-zingiberene (28.01%), *β*-sesquiphellandrene (18.09%), *β*-bisabolene (9.72%), *α*-farnesene (7.40%), and *β*-citral (5.48%), which represents about 68.70% of the total EO compounds. The findings agree with previous studies in ginger EO containing *α*-zingiberene and *β*-sesquiterpene as principal components (10%–60%) responsible for different bioactivities such as antioxidant and antimicrobial properties [32, 33]. They can be used as a natural, long-lasting food preservative. Similarly, citral is another precarious compound identified from ginger EO, which inhibits much bacterial and fungal growth by reducing ATPase interactions, mitochondrial structural alteration, and cell wall and membrane deterioration [34–36]. The chemical composition and its variations can be influenced by several factors, such as varieties, agroclimatic conditions, stage of maturity, the part where the EO is derived, and distillation conditions [37, 38].

The presence of 18 compounds was identified from clove oil, which covers 98.64% of the total EO composition (Table 1). Eugenol (70.12%) was identified as a major constituent, followed by trans-*β*-caryophyllene (12.56%) and eugenol acetate (11.32%). Similarly, other studies revealed that eugenol is the prime component in clove oil [39–41] and is used in food, medical, cosmetics, and agricultural industries as an antioxidant, antimicrobial, antiviral, and antifungal agent [42, 43]. The amount of eugenol in the EO can be directly related to the different geographic regions where the plant has grown, influencing biotic and abiotic factors such as seasonality, developmental stage, plant age, and climatic conditions [44]. In addition, the extraction method of EO can also influence the content of its volatile compounds [45], these differences in chemical composition can be directly related to pharmacological properties [46].

GC–MS results reveal that 12 components in eucalyptus EO are identified, accounting for 99.54% of the total EO composition (Table 2), which mainly consisted of oxygenated and nonoxygenated monoterpenes as well as oxygenated sesquiterpenes. Of these, 1,8-cineole, also named as eucalyptol (52.95%) and terpinen-4-ol (2.63%) were the main oxygenated monoterpenes, while *α*-pinene (20.75%), p-Cymene (10.10%), and *β*-myrcene (8.70%) were the main nonoxygenated monoterpenes. These results are similar to EOs obtained from Tanzania, Iran, and Tunisia [47–49]. Most of these compounds are commercially used in the food, pharmaceutical, agricultural, and chemical industries as flavourants, drugs, pesticides, and industrial feedstocks. Particularly, eucalyptol is used in flavourings, fragrances, and cosmetics due to its pleasant aroma and taste. It is also the main ingredient in mouthwashs, cough suppressants, insecticides, and insect repellents [47, 50]. In earlier studies, Tyagi and Malik [51] and Kumar et al. [52] reported 45.4% and 33.6% of eucalyptol, respectively, from eucalyptus EOs from India, which is less than our results. This reveals that the chemical composition of EOs could vary within India, due to the different geographic regions, which can influence the EO composition.

In garlic oil, 13 compounds were detected in GC–MS analysis, covering more than 96.15% of the total EO composition (Table 2). The chemical composition showed that allyl polysulfides and terpenes, such as diallyl disulfide (22.18%), diallyl trisulfide (43.25%), allyl methyl trisulfide (9.32%), and 2-vinyl-4H-1,3-dithiine (4.80%), were the most predominant components, and the similar result was observed by Herrera-Calderon et al. (2021). Most of these components are responsible for the characteristics of odour and flavour, antioxidant [53], antimicrobial [54], antifungal [55], anti-inflammatory [56], and antiobesity properties [57].

### 3.2. In Vitro Antioxidant Activities of EOs

#### 3.2.1. DPPH Radical Scavenging Assay

DPPH values of EOs with different concentrations showed a significant difference (*p* < 0.05) (Table 3). The inhibition percentage has gradually increased with the increasing concentration of EOs. Based on the results, it can be inferred that eucalyptus EO showed maximum antioxidant activity (93.36 ± 0.30%) followed by ginger (87.52 ± 2.84%) and garlic (67.14 ± 1.21%) at 100% concentration. At the same time, the clove EO showed 70.84 ± 3.95% inhibition even at a 5% level, which showed the predominant radical scavenging activity at lower concentrations than the other EOs. The chemical composition (Table 1) displayed the high contribution of eugenol (70.12%), indicating that the total antioxidant activity of the polyphenol mixture is a reflex of the individual components' activity [58, 59]. It allows hydrogen atom donation, stabilizes the phenoxyl radical generation, and creates stable compounds. Generally, eugenol reduces two or more DPPH radicals despite having only one hydrogen atom in the hydroxyl group. Further development of dehydrodieugenol (dimers) with two phenolic hydroxyl groups originating from eugenol intermediate radicals has also been proposed as a mechanism between eugenol and DPPH radicals [60]. Similarly, the level of antioxidant activity of other EOs, including eucalyptus, ginger, and garlic, are influenced by their principal constituents such as 1,8-cineole [61], *α*-zingiberene [62], and diallyl trisulfide [63], respectively. These compounds are responsible for stabilizing the free radicals by transferring hydrogen atoms or electrons, which can prevent the oxidation process [60, 64].

#### 3.2.2. FRAP Assay

The reducing power of EOs from ferric iron (Fe^3+^) to ferrous iron (Fe^2+^) was measured using the FRAP method, and the results are listed in Table 3. Among the EOs, ginger possesses maximum reducing potential (561.87 ± 8.13 *μ* mol Fe (II) g^−1^) followed by garlic (155.45 ± 1.12 *μ* mol Fe (II) g^−1^) and eucalyptus (87.62 ± 2.71 *μ* mol Fe (II) g^−1^) at 100%, whereas clove EO demonstrated powerful reducing ability (142.29 ± 1.05 *μ* mol Fe (II) g^−1^) even at a 5% level, surpassing the 100% eucalyptus EO. This evidence shows that the electron-donating properties of clove EO neutralize free radicals and form a stable product. Similarly, other research studies revealed that eugenol and other phenolic components are the predominant components in clove EO, responsible for its potential antioxidant properties [65, 66].

#### 3.2.3. TPC

Phenolic compounds generally have a positive correlation with antioxidant activities due to their electron-donating capability during free radical reactions, and results have shown an increasing trend with increasing concentrations (*p* < 0.05) (Table 3). The results indicated that clove EO contains high TPC (375.91 ± 14.21 mg/100 g) at a 5% level, directly correlated with high antioxidant activities. Other EOs showed less TPC than clove EO, even at 25% concentration. Among the other EOs, ginger showed high TPC (124.66 ± 6.03 mg/100 g), followed by garlic (102.33 ± 5.39 mg/100 g), and eucalyptus EO had the least TPC (19.41 ± 12.99 mg/100 g). Generally, TPC may be attributable to intrinsic and extrinsic factors, such as cultivars, soil types and growing conditions, maturity state, and harvesting conditions [67]. The results revealed that the TPC of EOs is directly correlated with antioxidative properties, which may help the cells protect against the oxidative damage produced by free radicals [68]. Moreover, these phenolic compounds are responsible for the defense mechanisms of EOs against microbial attack or environmental stress [69].

### 3.3. In Vitro Antimicrobial Activities of EOs

#### 3.3.1. Agar Well Diffusion Assay

**Figure 1:**
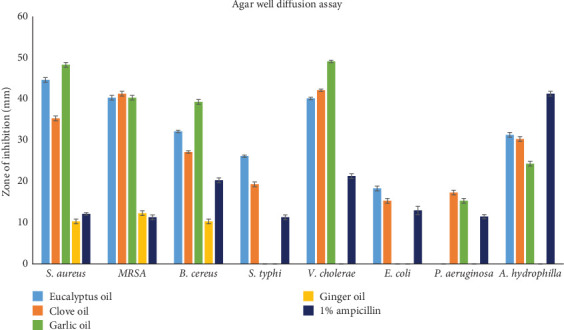
Zone of inhibition (millimeters) of different essential oils against foodborne pathogens.

**Figure 2:**
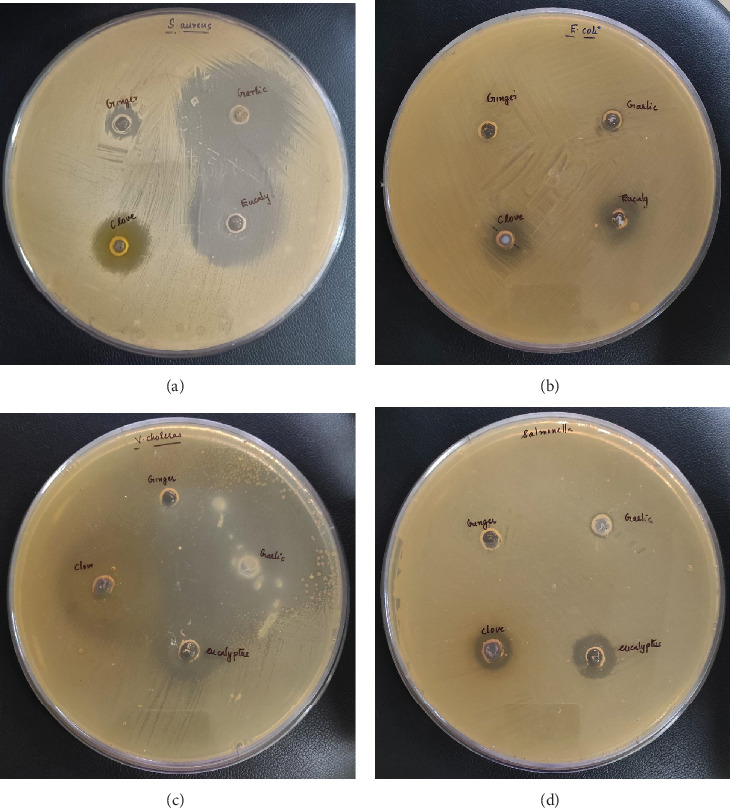
Essential oils showed the zone of inhibition (millimeters) against (a) *S. aureus*, (b) *E. coli*, (c) *V. cholera*, and (d) *S. typhi.*

It is used to study the antimicrobial properties of eucalyptus, clove, garlic, and ginger EOs against foodborne and fishborn bacteria (Table 4, Figures 1 and 2). Ginger EO displayed antibacterial activity against Gram-positive bacteria such as *S. aureus* (10.33 ± 0.57 mm), MRSA (12.33 ± 0.57 mm), and *B. cere*us (10.33 ± 0.57 mm). Still, it failed to inhibit the Gram-negative bacterial growth, possibly due to the thick outer lipopolysaccharide layer of Gram-negative bacteria structure, which provides additional protection against ginger antibacterial compounds and reduces the ginger EO activity [70, 71]. In contrast, Gram-positive bacteria have a monopetide layer [72], which may be easily ruptured by the ginger EO. Garlic EO exhibited the maximum zone of inhibition against both Gram-positive (*S. aureus*, 48.33 ± 0.57 mm) and negative bacteria (*V. cholerae*, 49.16 ± 0.28 mm). However, it showed no inhibition zone against *S. typhi* and *E. coli*. Organosulfur compounds such as allicin and other aliphatic sulfides are responsible for the antimicrobial properties of garlic oil [73]. These compounds protect the gut microbiome and prevent food poisoning [74, 75]. Similarly, eucalyptus EO indicated the highest inhibition zone against Gram-positive bacteria *S. aureus* (44.66 ± 0.57 mm) followed by Gram-negative bacteria *V. cholerae* (40.16 ± 0.28 mm). However, it did not exhibit antimicrobial activity against *P. aeruginosa*. This result agrees with a previous study [76], where the EO of eucalyptus showed better antimicrobial properties against gram-positive bacteria than gram-negative bacteria. The antimicrobial properties of eucalyptus EO have been due to their prime compounds, such as 1,8-cineole, *α*-pinene, p-Cymene, and *β*-myrcene [77], which affect the fatty acids in the bacterial cell membrane and cytoplasm, as well as proteins, ATP, cell morphology, and antiquorum sensing activities [78].

Moreover, clove EO exhibited a zone of inhibition against all the tested microorganisms. It showed the highest zone of inhibition against Gram-negative bacteria *V. cholerae* (42.16 ± 0.28 mm), followed by Gram-positive bacteria, MRSA (41.33 ± 0.57 mm). Several researchers have found clove effective against gram-positive and negative microorganisms [79–82]. Among the EOs, clove EO demonstrated the highest zone of inhibition against MRSA in the agar well diffusion method, revealing the potential antimicrobial effect against multidrug resistance organisms. In the present study, the higher concentration of eugenol, eugenyl acetate, and other phenolic compounds in clove EO is a significant attribute of its antimicrobial activity [83]. These compounds can denature proteins and change the cell permeability by reacting with cell membrane phospholipids, which cause the inhibition of both Gram-negative and Gram-positive bacteria growth [84, 85]. Based on the antibacterial activity, the hierarchy of the oils' has been observed as follows: garlic > eucalyptus > clove > ginger EO for the Gram-positive bacteria, garlic > clove > eucalyptus EO for Gram-negative bacteria. Moreover, the antimicrobial activity of EOs has correlated with the diffusion abilities such as diffusion coefficient, zeta potential, and droplet size of EOs through bacterial cell membranes [86]. Similarly, different factors, including microbial cultures, geographical origin, plant part, extraction methods, and harvest period, affect the antimicrobial activity of EOs [87]. Overall, the study demonstrated that EOs, except ginger, demonstrated broad-spectrum antibacterial properties against Gram-positive and Gram-negative bacteria. In particular, clove EO displayed a prominent antimicrobial effect against all the tested pathogens and was proposed as a broad-spectrum antimicrobial agent in pharmaceutical industries or as a biopreservative in the food or cosmetic industries.

#### 3.3.2. MIC and MBC Method

The antibacterial effect of EOs was determined using MIC and MBC methods, and the results are given in Table 4. There was a high correlation between the zone of inhibition and the MIC and MBC results of clove EO, which showed the largest zone of inhibition (*S. aureus*, MRSA, and *V. cholerae*) and lowest MIC and MBC values, ranging from 0.000089 ± 0.0002 to 2.94 ± 0.90 and 0.00018 ± 0.00005 to 2.94 ± 0.90 mg/mL, respectively. Other EOs showed little correlation between the zone of inhibition and MIC and MBC values, which showed the largest inhibition zone but not the lowest MIC and MBC values compared to clove EO. Eucalyptus EO showed the MIC and MBC values ranged from 1.33 ± 0.40 to 85.50 ± 0.80 and 5.34 ± 1.70 to 85.50 ± 0.80 mg/mL, respectively. Garlic oil displayed MIC and MBC values ranging from 0.005 ± 0.001 to 515.70 ± 0.00 and 5.14 ± 1.70 to 386.70 ± 12.80 mg/mL, respectively but did not show antimicrobial activity against *E. coli*. Similarly, ginger oil exhibited MIC and MBC values ranging from 5.34 ± 1.70 to 439.15 ± 0.00 mg/mL and 5.34 ± 1.70 to 439.15 ± 0.00 mg/mL, respectively, and did not show microbial resistance against salmonella and *E. coli*. A similar range was confirmed in another study against *S. aureus*, *B. subtilis*, *E. coli*, and *Penicillium* spp. [88]. This could be due to the EOs' relatively more water-soluble components that can diffuse further through the agar during overnight incubation. The lack of correlation between the results of EOs from the two assays suggests that the two methods are not necessarily comparable. The agar well diffusion method has been regarded as problematic for the antimicrobial testing of natural products, qualitatively, and useful for screening purposes only. Quantitative testing can be conducted using the broth microdilution method to study the antimicrobial activity [89]. Based on the MIC values, the hierarchy has been made for the EOs, that is, clove oil > eucalyptus oil > garlic > ginger (based on lower MIC value). In particular, clove oil demonstrated potent antibacterial activity against all the tested microorganisms. Also, it exhibited the maximum MIC and MBC values against MRSA, which indicates the antimicrobial potential against multidrug-resistant bacteria.

## 4. Conclusion

The current study demonstrated different EOs' antioxidant and antimicrobial properties and could be a potential natural antioxidant and antimicrobial source to replace the synthetic compounds. Regarding antimicrobial activities, eucalyptus, clove, and garlic EOs demonstrated their antimicrobial properties against gram-positive and gram-negative bacteria and could be used as natural preservatives to delay the bacterial spoilage of various food products. Among them, clove EO exhibited better antimicrobial activity against all the tested microorganisms, mainly showing a maximum zone of inhibition against MRSA, which reveals the potential against multidrug-resistant bacteria and makes these oils a possible alternative to synthetic antibiotics. Similarly, based on the antioxidant assay, clove oil possesses a high antioxidant capacity with the least concentration and can readily be used as a natural preservative to delay the oxidation process, maintain nutritional quality, and prolong the food product's shelf life. Further comprehensive research should be conducted to understand the underlying mechanism behind these major components' antioxidant and antimicrobial properties in EOs.

## Figures and Tables

**Table 1:** Chemical composition of ginger and clove essential oils.

**No.**	**Ginger oil**			**Clove oil**		
	**Compound name**	**RT (min)**	**% in sample**	**Compound name**	**RT (min)**	**% in sample**
1	*α*-Pinene	2.97	0.75	*α*-Pinene	7.554	0.02
2	Camphene	3.57	2.53	Camphene	8.074	0.04
3	*β*-Pinene	4.26	0.10	Linalool	13.325	0.04
4	*β*-Myrcene	5.64	0.70	Benzyl acetate	15.44	0.03
5	*α*-Limonene	6.55	0.69	2-Carbomethoxyphenol	16.443	0.12
6	1,8-Cineole	6.89	3.73	Eugenol	22.613	70.12
7	*α*-Terpinolene	9.04	0.14	*α*-Copaene	22.763	1.27
8	*α*-Copaene	15.25	0.41	Trans-*β*-caryophyllene	24.489	12.56
9	Camphor	15.72	0.09	*α*-Caryophyllene	25.664	1.64
10	*β*-Linalool	17.05	1.15	*δ*-Cadinene	26.339	0.04
11	*β*-Elemene	18.17	0.70	*γ*-Muurolene	26.461	0.04
12	*β*-Farnesene	20.69	0.32	Germacrene D	26.653	0.1
13	*β*-Citral	20.95	5.48	Zingiberene	27.095	0.07
14	*α*-Cadinene	21.25	0.29	Farnesene	27.523	0.23
15	*α*-Terpineol	21.58	2.52	*γ*-Cadinene	27.853	0.03
16	Borneol	21.56	1.95	*β*-Cadinene	28.177	0.33
17	Germacrene D	21.82	1.54	Eugenol acetate	28.637	11.32
18	*γ*-Cadinene	22.07	2.59	Caryophyllene epoxide	30.509	0.64
19	*α*-Eudesmene	22.42	0.80			
20	*α*-Zingiberene	22.63	28.01			
21	*β*-Bisabolene	22.80	9.72			
22	*α*-Farnasene	23.54	7.40			
23	*β*-Sesquiphellendrane	24.15	18.09			
24	*γ*-Elemene	25.52	0.16			
25	Germacrene B	25.70	0.30			
26	*β*-Geraniol	26.52	1.01			
27	Trans-nerolidol	33.01	1.66			
28	Elemol	34.01	0.30			
29	*γ*-Eudesmol	34.51	0.13			
30	*α*-Eudesmol	37.94	0.25			
31	*β*-Eudesmol	38.14	0.73			

**Table 2:** Chemical composition of eucalyptus and garlic essential oils.

**No.**	**Eucalyptus oil**			**Garlic oil**		
	**Compound name**	**RT (min)**	**% in sample**	**Compound name**	**RT (min)**	**% in sample**
1	*α*-Pinene	5.84	20.75	Allyl methyl disulfide	12.70	0.48
2	Camphene	6.20	0.53	3H-1,2-Dithiole	14.20	2.31
3	*β*-Pinene	6.82	0.01	Diallyl disulfide	18.10	22.18
4	*β*-Myrcene	7.17	8.70	1-Propenyl 2-propenyl-(E)-disulfide	18.70	1.02
5	p-Cymene	7.85	10.10	Allyl methyl trisulfide	20.05	9.32
6	Eucalyptol	7.98	52.95	3-Vinyl-1,2-dithiacyclohex-4-ene	21.70	2.60
7	*γ*-Terpinene	8.53	2.46	4H-1,2,3-Trithiine	22.20	2.97
8	Citronellal	10.22	1.20	2-Vinyl-4H-1,3-dithiine	22.40	4.80
9	Terpinen-4-ol	10.64	2.63	Diallyl trisulfide	24.95	43.25
10	*α*-Terpineol	10.87	0.10	1-Allyl-3-propyltrisulfane	25.20	1.57
11	Caryophyllene	14.19	0.10	5-Methyl-1,2,3,4-tetrathiane	27.22	1.65
12	Alloaromadendrene	14.45	0.01	Diallyl tetrasulfide	31.65	0.88
13				*α*-Bisabolol	35.45	3.12

**Table 3:** Antioxidant activities of essential oils.

**Essential oil**	**DPPH assay (% of inhibition)**				
**Conc. (** **v**/**v)**	**20%**	**40%**	**60%**	**80%**	**100%**
Ginger	23.68 ± 7.13^Ba^	43.94 ± 9.80^Bb^	65.40 ± 7.15^Bc^	73.64 ± 2.95^Bc^	87.52 ± 2.84^Bd^
Garlic	6.27 ± 1.37^Aa^	13.21 ± 1.59^Ab^	39.84 ± 1.08^Ac^	60.05 ± 1.62^Ad^	67.14 ± 1.21^Ae^
Eucalyptus	6.15 ± 0.89^Aa^	24.70 ± 2.34^Ab^	60.95 ± 2.00^Bc^	83.13 ± 3.60^Cd^	93.36 ± 0.30^Ce^
**Concentration**	**1%**	**2%**	**3%**	**4%**	**5%**
Clove	16.98 ± 2.19^a^	26.74 ± 2.53^b^	42.07 ± 2.44^c^	57.28 ± 2.94^d^	70.84 ± 3.95^e^

**FRAP assay (*μ* mol Fe (II) g** ^**−1**^ **)**					
**Conc. (** **v**/**v)**	**20%**	**40%**	**60%**	**80%**	**100%**
Ginger	136.62 ± 4.04^Ca^	165.45 ± 6.91^Cb^	385.12 ± 13.11^Cc^	497.87 ± 8.77^Cd^	561.87 ± 8.13^Ce^
Garlic	94.70 ± 4.41^Ba^	112.12 ± 3.22^Bb^	128.87 ± 0.80^Bc^	143.20 ± 7.18^Bd^	155.45 ± 1.12^Be^
Eucalyptus	43.95 ± 5.35^Aa^	60.87 ± 1.18^Ab^	70.87 ± 2.48^Ac^	76.45 ± 1.29^Ac^	87.62 ± 2.71^Ad^
**Conc. (** **v**/**v)**	**1%**	**2%**	**3%**	**4%**	**5%**
Clove	41.62 ± 2.68^a^	52.87 ± 0.97^b^	77.70 ± 1.31^c^	108.79 ± 1.86^d^	142.29 ± 1.05^e^

**Total phenolic content (mg phenols 100** g^**−1**^**)**					
**Conc. (** **v**/**v)**	**5%**	**10%**	**15%**	**20%**	**25%**
Ginger	7.41 ± 0.38^Ba^	13.5 ± 1.56^Aa^	53 ± 5.01^Bb^	91.41 ± 5.75^Cc^	124.66 ± 6.03^Cd^
Garlic	8.08 ± 0.57^Ba^	21.75 ± 6.08^Bb^	45.33 ± 5.34^Bc^	76.41 ± 4.91^Bd^	102.33 ± 5.39^Be^
Eucalyptus	5.16 ± 29.77^Aa^	8.33 ± 21.97^Ab^	11.83 ± 7.79^Ac^	14.75 ± 17.84^Ad^	19.41 ± 12.99^Ae^
**Concentration**	**1%**	**2%**	**3%**	**4%**	**5%**
Clove	50.08 ± 5.06^a^	104 ± 10.37^b^	169.41 ± 6.80^c^	248.33 ± 9.52^d^	375.91 ± 14.21^e^

*Note:* Results are mean ± standard deviation (*n* = 3); values with different letters within a row (a–e) and values with different letters within a column (A–C) are significantly different (*p* < 0.05) in one-way ANOVA followed by Duncan multiple range test.

Abbreviations: DPPH, 2,2-diphenyl-1-picrylhydrazyl; FRAP, ferric reducing antioxidant ability.

**Table 4:** MIC and MBC values of essential oils (milligrams per milliliter).

**Bacterial stains**	**Eucalyptus oil**		**Clove oil**		**Garlic oil**		**Ginger oil**	
	**MIC**	**MBC**	**MIC**	**MBC**	**MIC**	**MBC**	**MIC**	**MBC**
*S. aureus*	5.34 ± 1.7	10.69 ± 3.5	0.001 ± 0.0004	0.001 ± 0.0004	5.14 ± 1.7	5.14 ± 1.7	5.34 ± 1.7	5.34 ± 1.7
MRSA	2.67 ± 0.8	10.69 ± 3.5	0.000089 ± 0.0002	0.00018 ± 0.00005	0.005 ± 0.001	48.3 ± 1.7	10.29 ± 3.4	20.58 ± 6.8
*B. cereus*	85.5 ± 0.8	85.5 ± 0.8	0.0007 ± 0.0002	0.011 ± 0.003	6.04 ± 0.001	6.04 ± 1.7	20.58 ± 6.8	20.58 ± 6.8
*S. typhi*	42.78 ± 1.4	42.78 ± 1.4	0.001 ± 0.0004	1.472 ± 0.4	515.7 ± 0.00	—	—	—
*V. cholerae*	1.337 ± 0.4	5.34 ± 1.7	0.00018 ± 0.00005	0.0003 ± 0.0001	12.08 ± 4.0	386.7 ± 12.8	82.34 ± 3.4	82.34 ± 3.4
*E. coli*	85.5 ± 0.8	85.5 ± 0.8	2.944 ± 0.9	2.944 ± 0.9	*—*	—	—	—
*P. aeruginosa*	42.78 ± 1.4	85.5 ± 2.5	0.368 ± 0.1	1.472 ± 0.4	193.3 ± 4.4	—	439.15 ± 0.00	439.15 ± 0.00
*A. hydrophilla*	5.34 ± 1.7	42.78 ± 4.2	0.0007 ± 0.0002	0.002 ± 0.0009	48.3 ± 6.1	96.68 ± 3.2	82.34 ± 7.4	82.34 ± 7.4

*Note:* Results are shown as mean ± standard deviation (*n* = 3).

## Data Availability

The primary data used to support the findings of this study are available from the corresponding author upon request.
