# Supplementary material for: Biochemical, Antioxidant, and Antimicrobial Profiling of Essential Oils of Indian Origin for Culinary Applications
Source: Int J Food Sci. 2024 Dec 27;2024:9326683. doi: 10.1155/ijfo/9326683 (PMC11698606; doi:10.1155/ijfo/9326683)
Supplement: Supporting Information — Additional supporting information can be found online in the Supporting Information section. [file 9326683.f1.docx]

| **Sr. No.** | **Peer Reviewer comments** | **Answers** |
| --- | --- | --- |
| 1 | GENERAL COMMENTS: In my candid opinion, the paper is impressive provides a detailed and comprehensive study on the biochemical composition, antioxidant, and antimicrobial properties of four essential oils of Indian origin. The introduction is comprehensive provides solid background justification for the study. The objectives are clear as well as the methodology are well-defined and clearly defined respectively. **The major weakness of the paper has do with language issue which needs to undergo thorough proofreading to correct grammar. Moreover the abstract is too detailed and needs to be summarized to present only major finding of the study.** | Corrected the grammar part wherever required in the manuscript  Modified the abstract slightly. |
| 2 | ADDITIONAL REQUESTS/SUGGESTIONS:  **The abstracts will need to be summarised to provide only key findings of the study. The paper also need to be thoroughly proofread to improve the grammar of the paper.** Generally the paper in overal is comprehensive and innovative. The paper provided a detailed comprehensive study on the biochemical composition, antioxidant and antimicrobial properties of essentials oils from Indian origin. The introduction is very comprehensive and exhaustive on the topic. The objectives are well defined and outlined. The method is also detailed and comprehensive that can be replicated. **The major set back of the paper has to do language infractions appearing all over the entire paper and needs to be worked on prior to publication.** | The abstract is summarised with key findings.  Improved the grammar of the paper. |
| 3 | ADDITIONAL REQUESTS/SUGGESTIONS:  **The abstract is too detailed and needs to be summarized to provide only the key findings of the study. The paper also needs to undergo thorough proofreading to comphensively correct the grammar prior to acceptance.**The paper is very impressive and provides valuable information that contribute scientific knowledge and solve real problems on the subject matter. The authord did well by providing a comprehensive study on the biochemical composition, antioxidant and antimicrobial properties of essential oils from Indian origins. The major weakness of this study is the language infractions that ought to be corrected prior to acceptance. | The abstract is summarised with key findings.  Improved the grammar of the paper. |
| 4 | ADDITIONAL REQUESTS/SUGGESTIONS:  Abstract should be summarized to provide only key findings of the study. The entire paper needs to undergo thorough proofreading to correct the overwhelming grammartical errors in spread accross the entire paper." | The abstract is summarised with key findings.  Improved the grammar of the paper. |
